# Efficient and practical synthesis of monoalkyl oxalates under green conditions†

**DOI:** 10.1039/d2ra04419f

**Published:** 2022-09-12

**Authors:** Tatiana Barsukova, Takeyuki Sato, Haruki Takumi, Satomi Niwayama

**Affiliations:** Graduate School of Engineering, Muroran Institute of Technology 27-1, Mizumoto-cho Muroran Hokkaido Japan niwayana@mmm.muroran-it.ac.jp

## Abstract

Monoalkyl oxalates are among the most important building blocks being applied to the synthesis of a variety of significant classes of compounds or applied to various cutting-edge reactions. However, their commercial availability is limited. Their synthetic methods are also limited because of the difficulty to synthesize them, and those hitherto reported are carried out in organic solvents often with the use of toxic reagents with mostly low to modest yields. Here we have developed practical synthesis of monoalkyl oxalates in aqueous media by applying the highly efficient selective monohydrolysis reactions of symmetric diesters which we reported previously. The best conditions apply an aqueous NaOH solution with relatively nontoxic THF or acetonitrile as a co-solvent at around 0–5 °C. The procedures are simple and environmentally friendly without requiring toxic or expensive reagents, yet yielding the corresponding half-esters in high yields with high purities. All the half-esters prepared here are stable over a long period of time. Therefore, our studies are expected to offer practical green methods for the synthesis of monoalkyl oxalates.

## Abstract 2

Simple and environmentally-friendly methods have been developed for the synthesis of monoalkyl oxalates without requiring toxic or expensive reagents.

## Graphical abstract

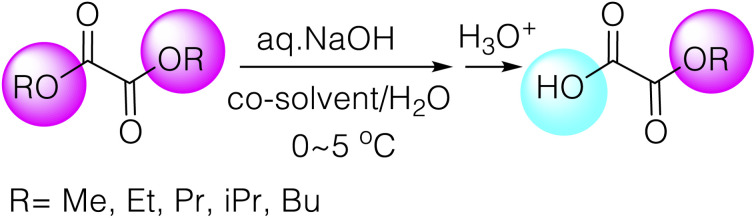

## Introduction

Half-esters are very important building blocks for organic synthesis, which have been applied to a wide range of significant classes of organic compounds such as pharmaceuticals and natural products as well as polymers.^1^ Among them, oxalate half-esters and their derivatives represent a unique structural unit with no carbon unit between the ester group and the carboxy group, and therefore are among the smallest building blocks in organic synthesis. They are still very important building blocks for the synthesis of a wide range of significant compounds.^2^ For example, (−)- and ent-(+)-Vindolin and related alkaloids were synthesized through an intermediary oxadiazole unit from an oxalate half-ester.^3^ Oxalate half-esters can serve as short linkers for the synthesis of a variety of pharmaceuticals.^4^ These oxalyl linkers have been found to play pivotal roles in the biochemical and biophysical assays, providing a suitable base for further optimization. Moreover, a fair number of other structure-activity relationship (SAR) studies including oxalates have also been reported for a variety of biological activities. For example, several cephalotaxine esters were synthesized for their antitumor activities.^5^ The synthesis of some analogues of 5, 8, 10-trideazafolate, which can serve as potential inhibitors of GAR Tfase or AICAR Tfase have also been reported.^6^ The SAR studies of synthetic derivatives of a natural product, narciclasine, isolated from *Narcissus* sp*.* have also been reported for anti-neoplastic activities.^7^ In addition, because of the structures, oxalate half-esters are susceptible to radical decarboxylation and deoxygenations. This characteristic has been applied to a variety of reactions with the use of oxalate half-esters or their derivatives, such as alkoxycarbonylation of heteroaromatic bases, deoxygenation of tertiary or secondary alcohols producing hydrocarbons, alkyl halides, *etc.*^8^ Such decarboxylation and deoxygenation reactions can also be followed by coupling reactions with aryl halides or by addition reactions to alkenes or Michael acceptors,^9^ which are also further applied to the synthesis of various complex natural products.^10^

**Scheme 1:**
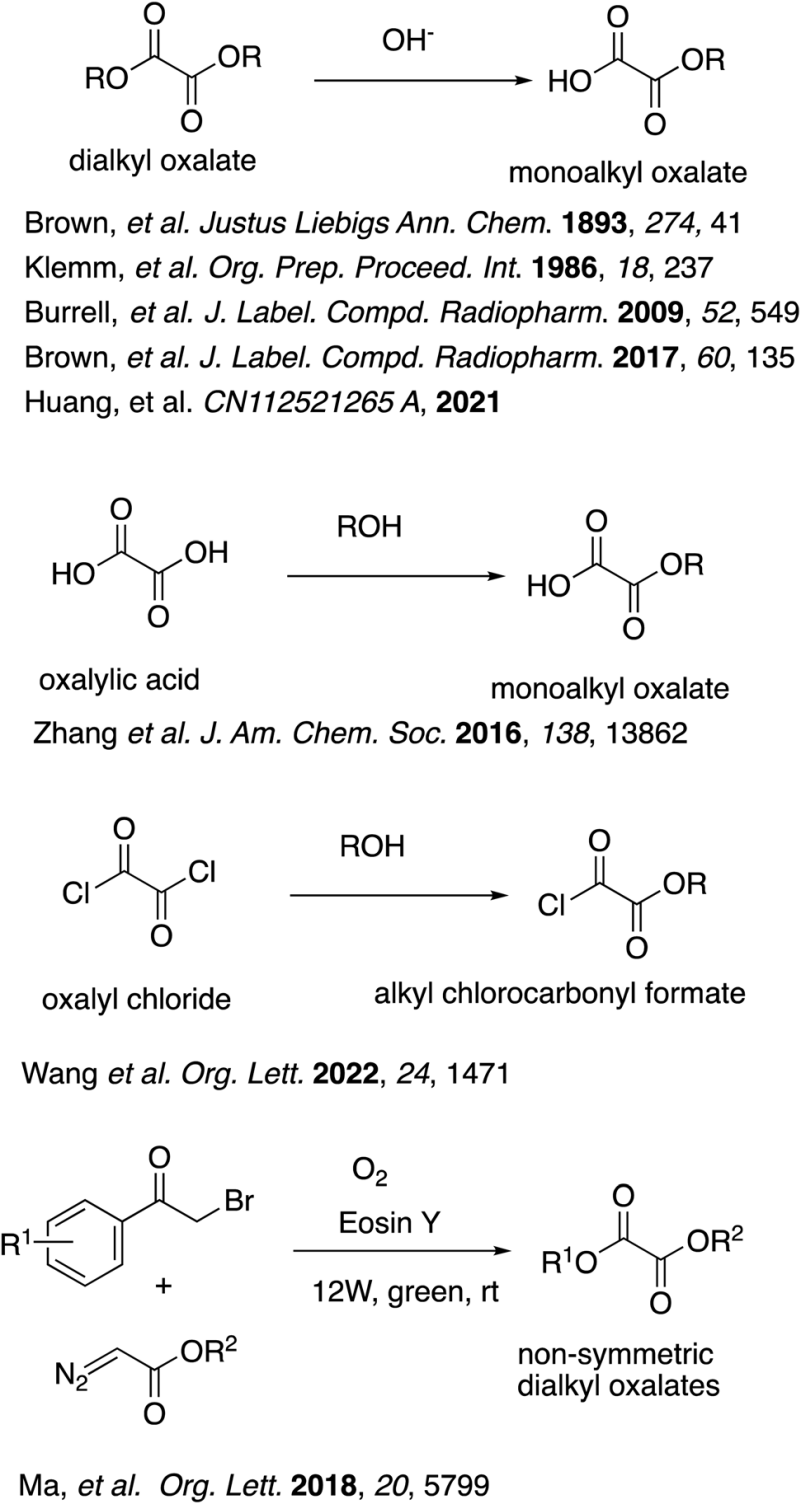
Synthesis of oxalate half-esters and their derivatives in the past.

Despite such enormous versatility, the commercial availability of oxalate half-esters is rather limited. Furthermore, the synthesis of oxalate half-esters is not necessarily easy because of the potential decarboxylation. Among the most typical and classical synthetic methods are partial hydrolysis or saponification of dialkyl oxalates,^11^ but the yields are usually low to modest (Scheme 1). Some oxalate half-esters and their derivatives were prepared by partial esterification of oxalic acid or oxalyl chloride as intermediates without further purification.^9*a*,*c*,12^ As for derivatives of oxalate half-esters, the synthesis of various non-symmetric dialkyl oxalates were reported from α-bromoketones and diazoacetates mediated by molecular oxygen and visible light in 50–90% yields.^13^ However, these reactions must be carried out in organic solvents, which can be of environmental concern. Some of them utilize toxic reagents such as oxalyl chloride, which generates corrosive HCl gas upon reaction with water.

**Scheme 2:**
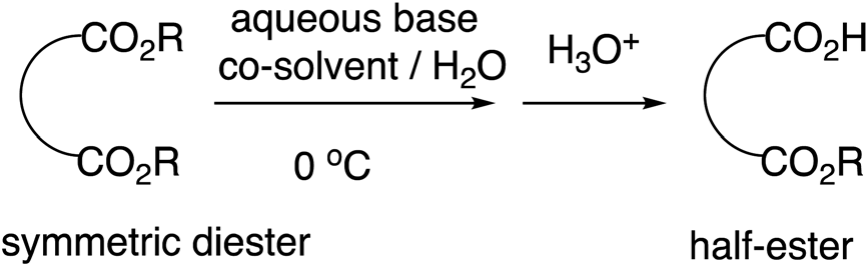
Selective monohydrolysis of symmetric diesters.

We previously reported highly efficient and practical selective monohydrolysis reactions of symmetric diesters in aqueous media (Scheme 2).^14^ These reactions selectively hydrolyzes only one of the two identical ester groups under mild and environmentally benign conditions, affording the corresponding half-esters in high yields in many cases. The procedures are straightforward; an aqueous base such as an aqueous NaOH or KOH solution is added dropwise to the starting diesters suspended in water that may contain a co-solvent when it facilitates the reactions at around 0 °C. The starting symmetric diesters can also be prepared inexpensively on a large scale or commercially available at a low cost.

According to our mechanistic hypothesis, once one of the two ester groups is monohydrolyzed, the formed monocarboxylates assemble micellar aggregates in which the hydrophilic carboxylates are directed outside and the remaining hydrophobic ester groups are directed inside, inhibiting further hydrolysis. Concordantly with this hypothesis, we found that a polar aprotic co-solvent such as THF and acetonitrile helps enhance the reaction rates and the selectivities, while a protic co-solvent such as an alcohol lowers the selectivity conceivably by dissociating the intermediary micellar aggregates.^15^ We had found that acetonitrile enhances reaction rates more than THF. In addition, we had reported that the reactivities somewhat differ depending on the type of the aqueous base with the order of KOH > NaOH > LiOH, and therefore KOH sometimes exhibits enhanced reactivities compared to NaOH, probably because of the stronger affinity of K^+^ with the oxygen atom of the carbonyl group, resulting in the enhanced electrophilicity of the starting ester carbonyl groups.^16^ The monohydrolysis reactions were found to show particularly high selectivities and often near-quantitative for symmetric cyclic 1,2-diesters, perhaps because of the contribution of the non-covalent interaction between the two properly close carbonyl groups referred to as *n* → π* interaction.^17^ However, on the basis of our mechanistic hypothesis, we previously reported that this selective monohydrolysis can also yield a variety of half-esters from other kinds of symmetric diesters such as malonates and their derivatives in greater than 95% yields, even allowing a mole-scale production, despite the fact that they easily undergo decarboxylation.^18^ By applying this selective monohydrolysis reaction, we now report efficient and practical synthesis of various half-esters of oxalates under simple and environmentally-friendly conditions.

## Results and discussion

Monomethyl oxalate is the simplest but among the most widely utilized monoalky oxalate. However, the commercial sources are limited. In order to synthesize monoalkyl oxalates, when we first tried selective monohydrolysis of dimethyl oxalate, 1, following the reaction conditions we initially reported^14^ and those that worked out the best for the selective monohydrolysis of dimethyl malonate using KOH as a base,^18^ only a trace amount of half-ester was obtained. The reaction proceeded extremely quickly, ending in less than a minute. Since the starting dimethyl oxalate itself is also miscible with water, although the reaction in water only without a co-solvent was acceptable (Table 1, entry 1), we adjusted the miscibility by using THF as a co-solvent and changed the base to one equivalent of an aqueous NaOH to reduce the reaction rate, and titrated the amount of the co-solvent as well as the reaction time as in Table 1. From this result, it was found that NaOH as a base and 5% of THF as a co-solvent for 10 minutes at around 0 °C worked out the most practical and perhaps the best from the economical points of view (Table 1, entry 4), although increasing the proportion of THF to 24% led to slightly better yield (Table 1, entry 12). Changing the co-solvent to other polar aprotic solvents, such as acetonitrile or acetone did not improve the yield. Cooling the reaction mixture to a little below 0 °C led to freeze of the reaction mixture and did not improve the yield either (data not shown).

**Table 1:** Selective monohydrolysis of dimethyl and diethyl oxalates

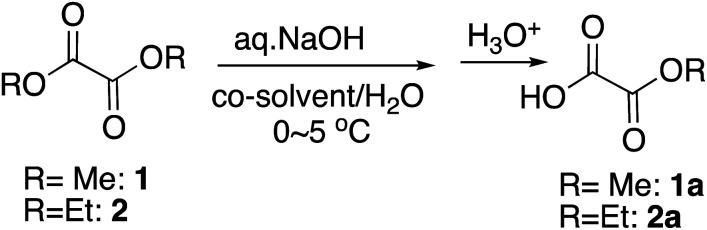					
Entry	R	Time (min)	Co-solvent	Co-solvent vol%	Yield^a^ (%)
1	Me	10	None	0	84
2		10	THF	1	84
3		10	THF	2.5	84
4		10	THF	5	90
5		10	CH_3_CN	5	80
6		10	Acetone	5	85
7		3	THF	5	77
8		3	THF	8	81
9		3	THF	10	88
10		3	THF	15	84
11		3	THF	20	87
12		3	THF	24	93
13		3	THF	30	89
14		3	Acetone	20	89
15	Et	20	None	0	61
16		10	THF	5	68
17		20	THF	5	90
18		20	THF	10	87
19		12	THF	30	92
20		10	CH_3_CN	5	61
21		10	Acetone	5	65

a Isolated yields after purification by column chromatography.

Next we tried selective monohydrolysis of diethyl oxalate, 2. Due to the increased size of the alkyl group, it was somewhat more resistant to the hydrolysis, and the yield was low without a co-solvent (Table 1, entry 15) as we had found before.^19^ The use of a co-solvent more obviously facilitated the reaction because of the slightly increased bulkiness, and essentially the same condition as above using one equivalent of an aqueous NaOH and THF as a co-solvent and 20 minutes of the reaction time turned out to be the most practical and the best conditions, yielding 90% of the half-ester (Table 1, entry 17), although again increasing the proportion of THF to 30% slightly raised the yield (Table 1, entry 19).

It is interesting to note that for the monohydrolysis of the above dimethyl and diethyl oxalates, 1 and 2, slightly adjusted procedures, by quickly adding a THF solution of the starting diester to a chilled water in ice-water bath, rather than adding cooled water to the THF solution of the diesters, followed by the addition of the aqueous base led to slightly better yield of the corresponding half-esters, 1a and 2a. This outcome is probably because this way is likely to help minimize the background hydrolysis by water.

For the selective monohydrolysis of diisopropyl oxalate, 3, the isopropyl group is bulkier than the ethyl group, and therefore under the same conditions as above, it was more resistant to the hydrolysis. Increasing the proportion of the co-solvent THF from 5% to 10% improved the yield of the half-ester (Table 2, entry 4) by increasing the contact of the isopropyl group and the reaction mixture as in the monohydrolysis of several bulky diesters we reported before.^19^ Here in the procedures, the starting diester was dissolved in THF at 0 °C and chilled water, and the base was added to this mixture as is customary.

**Table 2:** Selective monohydrolysis of other dialkyl oxalates

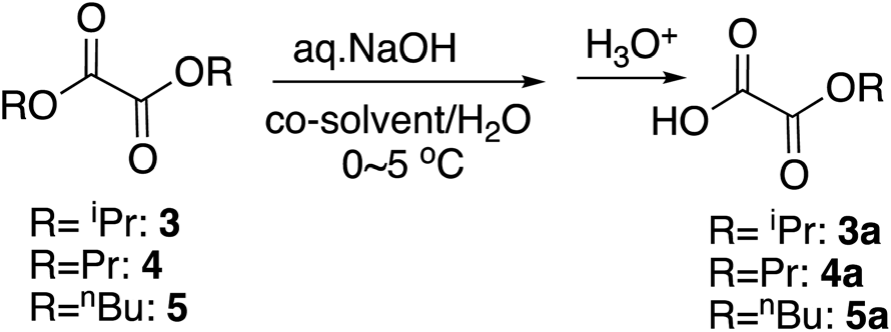					
Entry	R	Time (min)	Co-solvent	Co-solvent vol%	Yield^a^ (%)
1	^i^Pr	20	THF	5	83
2		20	CH_3_CN	5	64
3		20	THF	10	81
4		13	THF	10	85
5		5	THF	10	78
6		10	CH_3_CN	10	79
7		10	THF	20	81
8		5	CH_3_CN	20	75
9	Pr	20	THF	5	60
10		20	CH_3_CN	5	65
11		10	CH_3_CN	8	77
12		13	THF	10	75
13		13	CH_3_CN	10	80
14		10	THF	20	71
15		5	THF	13	81
16		5	CH_3_CN	20	79
17	^*n*^Bu	30	THF	5	21
18		30	THF	13	33
19		30	THF	21	58
20		30	THF	31	59
21		30	THF	42	68
22		40	THF	42	83
23		40	CH_3_CN	40	71
24		50	THF	42	69

a Isolated yields after purification by column chromatography.

We next studied the selective monohydrolysis of dipropyl oxalate, 4. Although increasing the proportion of THF helped improve the yields as well (Table 2, entry 15), changing the co-solvent from THF to acetonitrile, which was previously found to better increase the reaction rate as noted above, helped the outcomes to a similar extent (Table 2, entry 13). Unfortunately, use of aqueous KOH as a base or use of DMSO as the co-solvent decreased the yields probably due to the overreaction (data not shown).

For the selective monohydrolysis of dibutyl oxalate, 5, the butyl group is even bulkier than the propyl or isopropyl group. Therefore, we anticipated that greater proportion of co-solvent would be needed for comparable reactivities. When the proportion of THF was titrated as 9, 13, 25, 38, and 42% maintaining the reaction time to be 30 minutes, the best yield was observed when the proportion was 42% (Table 2, entry 21). Therefore, when the reaction time was increased to 40 minutes, the best yield was achieved as 83% (Table 2, entry 22). On the other hand, use of acetonitrile as the co-solvent gave comparable to lower yields (Table 2, entry 23). Changing the aqueous base from NaOH to KOH also lowered the yield (maximum 75%, data not shown) and did not help either.

## Conclusions

In summary, we have found the highly efficient and practical reaction conditions using an aqueous NaOH with THF or CH_3_CN, which are relatively nontoxic, as a co-solvent for selective monohydrolysis of various symmetric dioxalates producing the corresponding monoalkyl oxalates in high yields based on our mechanistic hypothesis. Unlike classical monosaponification reactions, which tend to produce yellowish complex mixtures, only the pure half-esters, the starting diesters, and the corresponding diacids, if they existed, were detected in all the cases we tried. To our knowledge, the yields here are among the highest hitherto reported, yet the procedures are extremely simple and environmentally benign without requiring toxic or expensive reagents. All the half-esters obtained here exhibited very high purities, and they remained stable over a long period of time. As monoalkyl oxalates are among the most widely utilized half-esters, considering the practicability and greenness, the synthetic versatility of this reaction is expected.

## Experimental

### General

The ^1^H NMR and ^13^C NMR spectra were recorded in CDCl_3_ with the use of a JEOL JNM-ECA500 spectrometer operated at 500 MHz for ^1^H NMR and 125 MHz for ^13^C NMR with tetramethylsilane (TMS) as an internal standard. The IR spectra were recorded on a JASCO 4100 FT-IR spectrometer.

### Typical procedures

#### Monohydrolysis of dimethyl oxalate

Dimethyl oxalate (0.184 g, 1.6 mmol) was dissolved in 1 mL of THF, and added to 13 mL of chilled water. The reaction mixture was immersed in an ice-water bath, and 6.5 mL of chilled 0.25 M aqueous NaOH was injected when the temperature of the reaction mixture reached 0–5 °C. After 10 minutes of stirring, the reaction mixture was acidified with 2 M HCl to pH 0.5–0.7, and extracted with approximately 10–15 mL of ethyl acetate four times. The extract was dried over anhydrous Na_2_SO_4_ and concentrated *in vacuo*. The crude product was purified by column chromatography with hexane and ethyl acetate.

##### Monomethyl oxalate (1a)

^1^H NMR (500 MHz, CDCl_3_) *δ* = 3.97 (s, 3H, OCH_3_), 10.15 (b.s, 1H, OH). ^13^C NMR (125 MHz, CDCl_3_) *δ* = 54.48, 158.29, 158.50. IR (neat, cm^−1^): 3203, 2923, 1738, 1161. HRMS Calcd for C_3_H_3_O_4_(M–H)^−^: 103.00259. Found: 103.00240. Rf: 0.1 (hexane : ethyl acetate = 1 : 1).

#### Monohydrolysis of diethyl oxalate

Diethyl oxalate (0.228 g, 1.6 mmol) was dissolved in 1 mL of THF, and added to 13 mL of chilled water. The reaction mixture was immersed in an ice-water bath, and 6.5 mL of chilled 0.25 M aqueous NaOH was injected when the temperature of the reaction mixture reached 0–5 °C. After 20 minutes of stirring the reaction mixture was acidified with 2 M HCl to pH 0.5–0.7, and extracted with approximately 10–15 mL of ethyl acetate four times. The extract was dried over anhydrous Na_2_SO_4_ and concentrated *in vacuo*. The crude product was purified by column chromatography with hexane and ethyl acetate.

##### Monoethyl oxalate (2a)

^1^H NMR (500 MHz, CDCl_3_) *δ* = 1.42 (t, 3H, CH_3_, *J* = 7.2 Hz), 4.42 (q, 2H, OCH_2_, *J* = 7.2 Hz), 10.09 (b.s, 1H, OH). ^13^C NMR (125 MHz, CDCl_3_) *δ*= 13.79, 64.24, 158.19, 159.27. IR (neat, cm^−1^): 3222, 2924, 1739, 1192. Anal. calcd. for C_4_H_6_O_4_: C, 40.68; H, 5.12. Found: C, 40.66; H, 5.12. Rf: 0.1 (hexane : ethyl acetate = 1 : 1).

#### Monohydrolysis of diisopropyl oxalate

Diisopropyl oxalate (0.272 g, 1.6 mmol) was dissolved in 2 mL of THF, and 12 mL of chilled water was added. The reaction mixture was immersed in an ice-water bath, and 6.5 mL of chilled 0.25 M aqueous NaOH was injected when the temperature of the reaction mixture reached 0–5 °C. After 13 minutes of stirring the reaction mixture was acidified with 2 M HCl to pH 1, and extracted with approximately 10–15 mL of ethyl acetate four times. The extract was dried over anhydrous Na_2_SO_4_, and concentrated *in vacuo*. The crude product was purified by column chromatography with hexane and ethyl acetate.

##### Monoisopropyl oxalate (3a)

^1^H NMR (500 MHz, CDCl_3_) *δ*= 1.39 (d, 6H, CH_3_, *J* = 6.3 Hz), 5.21 (sept., 1H, OCH, *J* = 6.3 Hz), 8.99 (b.s, 1H, OH). ^13^C NMR (125 MHz, CDCl_3_) *δ* = 21.44, 73.25, 157.58, 158.58. IR (neat, cm^−1^): 3135.69, 2988.16, 1730.80, 1181.19, 1097.30. Anal. calcd for C_5_H_8_O_4_: C, 45.46; H, 6.10. Found: C, 45.03; H, 6.08. Rf: 0.1 (hexane : ethyl acetate = 1 : 1).

#### Monohydrolysis of dipropyl oxalate

Dipropyl oxalate (0.272 g, 1.6 mmol) was dissolved in 2 mL of CH_3_CN, and 12 mL of chilled water was added. The reaction mixture was immersed in an ice-water bath, and 6.5 mL of chilled 0.25 M aqueous NaOH was injected when the temperature of the reaction mixture was reached 0–5 °C. After 13 minutes of stirring, the TLC indicated consumption of the starting diester and the reaction mixture was acidified with 2 M HCl to pH 1, and extracted with approximately 10–15 mL of ethyl acetate four times. The extract was dried over anhydrous Na_2_SO_4_, and concentrated *in vacuo*. The crude product was purified by column chromatography with hexane and ethyl acetate.

##### Monopropyl oxalate (4a)

^1^H NMR (500 MHz, CDCl_3_) *δ*= 0.99 (t, 3H, CH_3_, *J* = 7.4 Hz), 1.78 (m, 2H, CH_2_), 4.30 (t, 2H, OCH_2_, *J* = 6.7 Hz), 10.33 (b.s, 1H, OH). ^13^C NMR (125 MHz, CDCl_3_) *δ* = 10.24, 21.71, 69.73, 158.06, 158.79. IR (neat, cm^−1^): 3194.51, 2883.06, 1732.73, 1180.22. Anal. calcd for C_5_H_8_O_4_: C, 45.46; H, 6.10. Found: C, 45.27; H, 6.15. Rf: 0.1 (hexane : ethyl acetate = 1 : 1).

#### Monohydrolysis of dibutyl oxalate

Dibutyl oxalate (0.327 g, 1.6 mmol) was dissolved in 16 mL THF, and 16 mL of chilled water was added. The reaction mixture was immersed in an ice-water bath, and 6.5 mL of chilled 0.25 M aqueous NaOH was injected when the temperature of the reaction mixture reached 0–5 °C. After 40 minutes of stirring the reaction mixture was acidified with 2 M HCl to pH 1, and extracted with approximately 10–15 mL of ethyl acetate four times. The extract was dried over anhydrous Na_2_SO_4_ and concentrated *in vacuo*. The crude product was purified by column chromatography with hexane and ethyl acetate.

##### Mono-*n*-butyl oxalate (5a)

^1^H NMR (500 MHz, CDCl_3_) *δ*= 0.96 (t, 3H, CH_3_, *J* = 7.3 Hz), 1.44 (m, 2H, CH_2_), 1.75 (m, 2H, CH_2_), 4.35 (t, 2H, OCH_2_, *J* = 6.7 Hz), 9.57 (b.s, 1H, OH). ^13^C NMR (125 MHz, CDCl_3_) *δ*= 13.56, 18.92, 30.17, 67.92, 158.09, 159.46. IR (neat, cm^−1^): 3192.58, 2962.13, 1733.69, 1177.33. Anal. calcd for C_6_H_10_O_4_: C, 49.31; H, 6.90. Found: C, 49.74; H, 6.90. Rf: 0.1 (hexane : ethyl acetate = 1 : 1).

## Conflicts of interest

There are no conflicts to declare.

## Supplementary Material

RA-012-D2RA04419F-s001
