# Supplementary material for: Efficient and practical synthesis of monoalkyl oxalates under green conditions
Source: RSC Adv. 2022 Sep 12;12(39):25669–74. doi: 10.1039/d2ra04419f (PMC9465637; doi:10.1039/d2ra04419f)
Supplement: RA-012-D2RA04419F-s001 [file RA-012-D2RA04419F-s001.pdf]

This journal is © The Royal Society of Chemistry 2022

Electronic Supplementary Information

Efficient and Practical Synthesis of Monoalkyl Oxalates under Green Conditions

Tatiana Barsukova, Takeyuki Sato, Haruki Takumi, and Satomi Niwayama*

Graduate School of Engineering, Muroran Institute of Technology

27-1, Mizumoto-cho, Muroran, Hokkaido, Japan

E-mail: niwayana@mmm.muroran-it.ac.jp

Table of Contents

^{1}H NMR and ^{13}C NMR of the half-esters S2

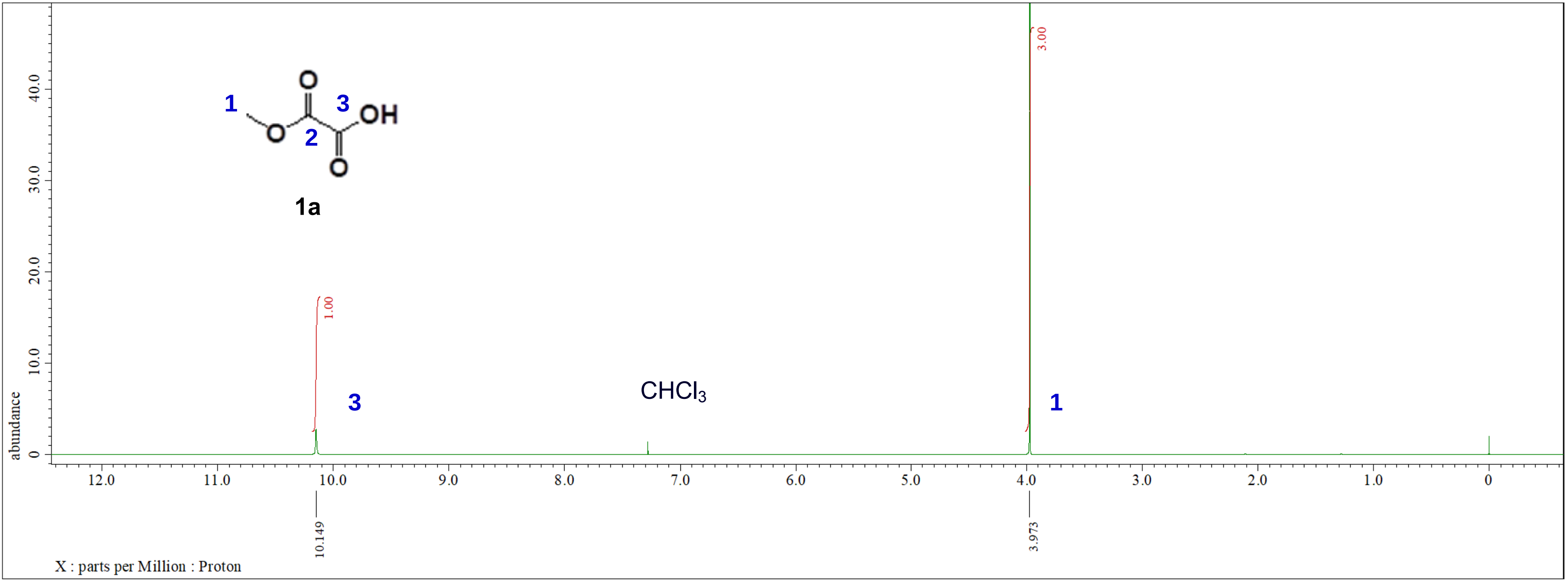
1
3
OH
2
O
O
O
1a
3.00
1.00
3
CHCl3
1
abundance
40.0
30.0
20.0
10.0
0
12.0
11.0
10.0
9.0
8.0
7.0
6.0
5.0
4.0
3.0
2.0
1.0
0
10.149
3.973
X : parts per Million : Proton

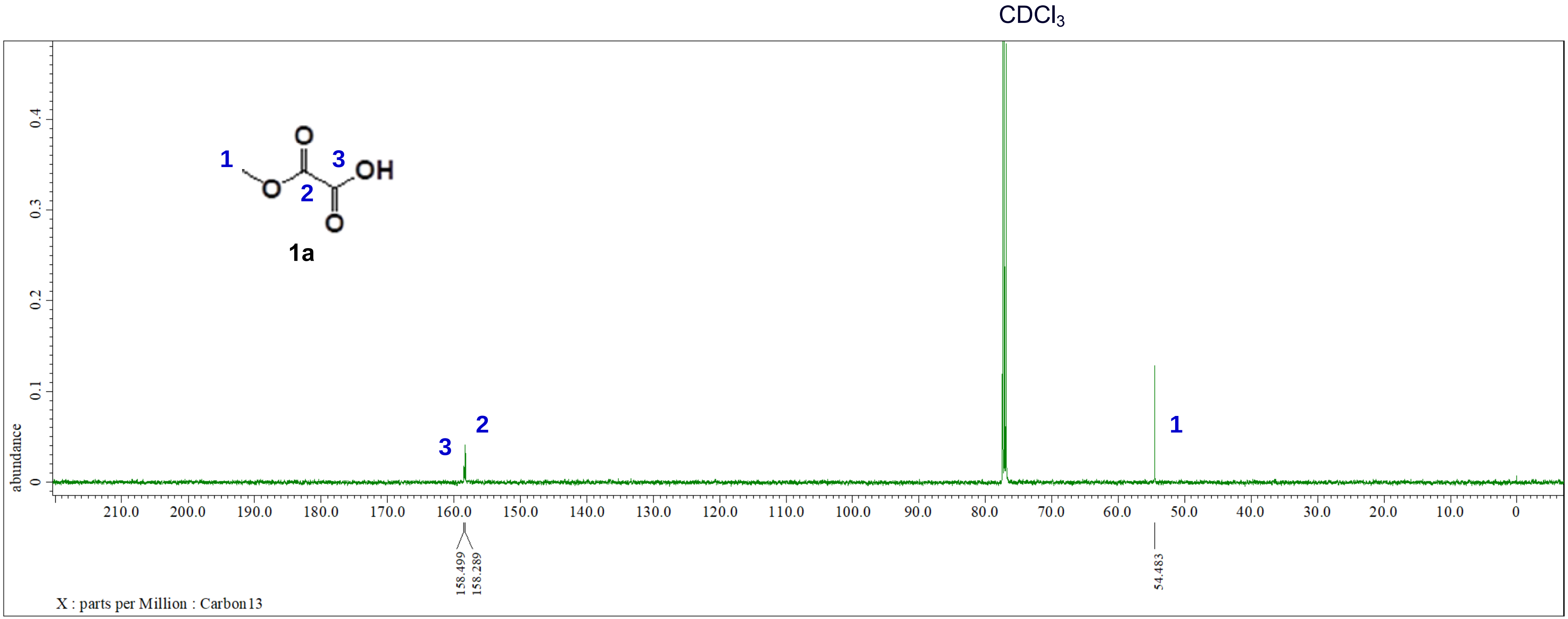
CDCl$_3$
1a
1
2
3
OH
abundance
0.4
0.3
0.2
0.1
0
210.0
200.0
190.0
180.0
170.0
160.0
150.0
140.0
130.0
120.0
110.0
100.0
90.0
80.0
70.0
60.0
50.0
40.0
30.0
20.0
10.0
158.499
158.289
54.483
X : parts per Million : Carbon13

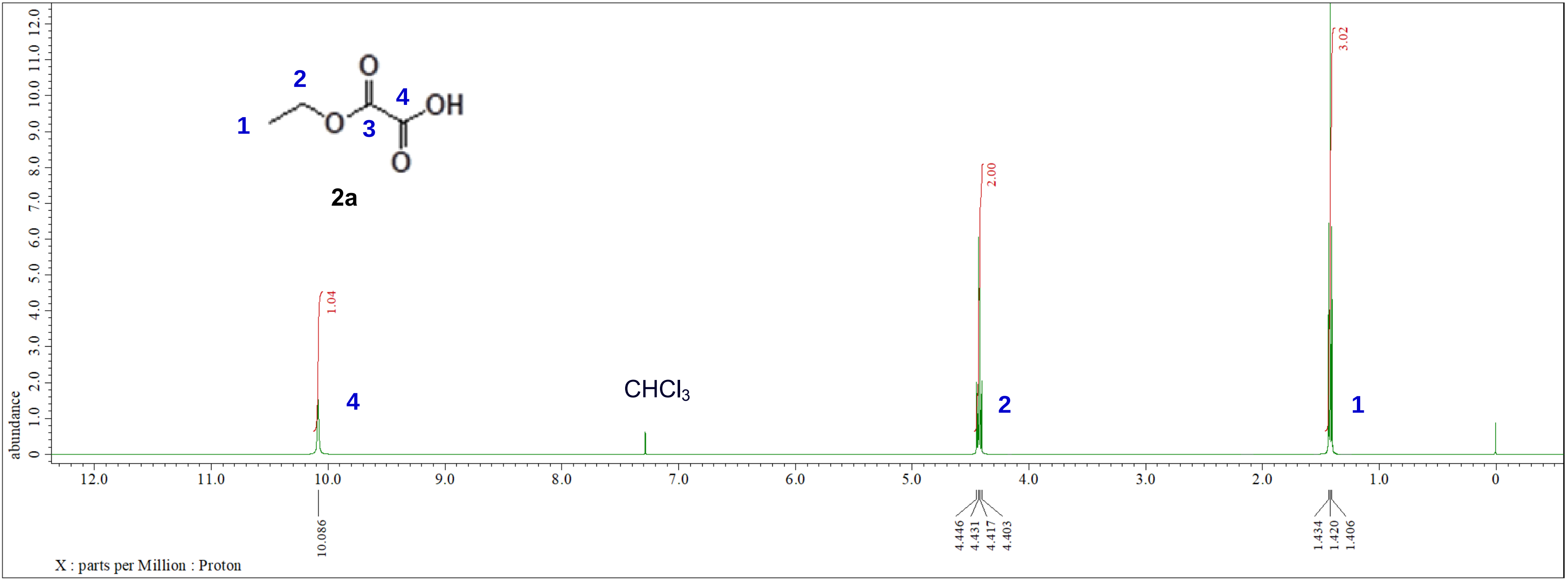

2a
CHCl3
1.04
2.00
3.02
10.086
4.446
4.431
4.417
4.403
1.434
1.420
1.406
abundance
X : parts per Million : Proton

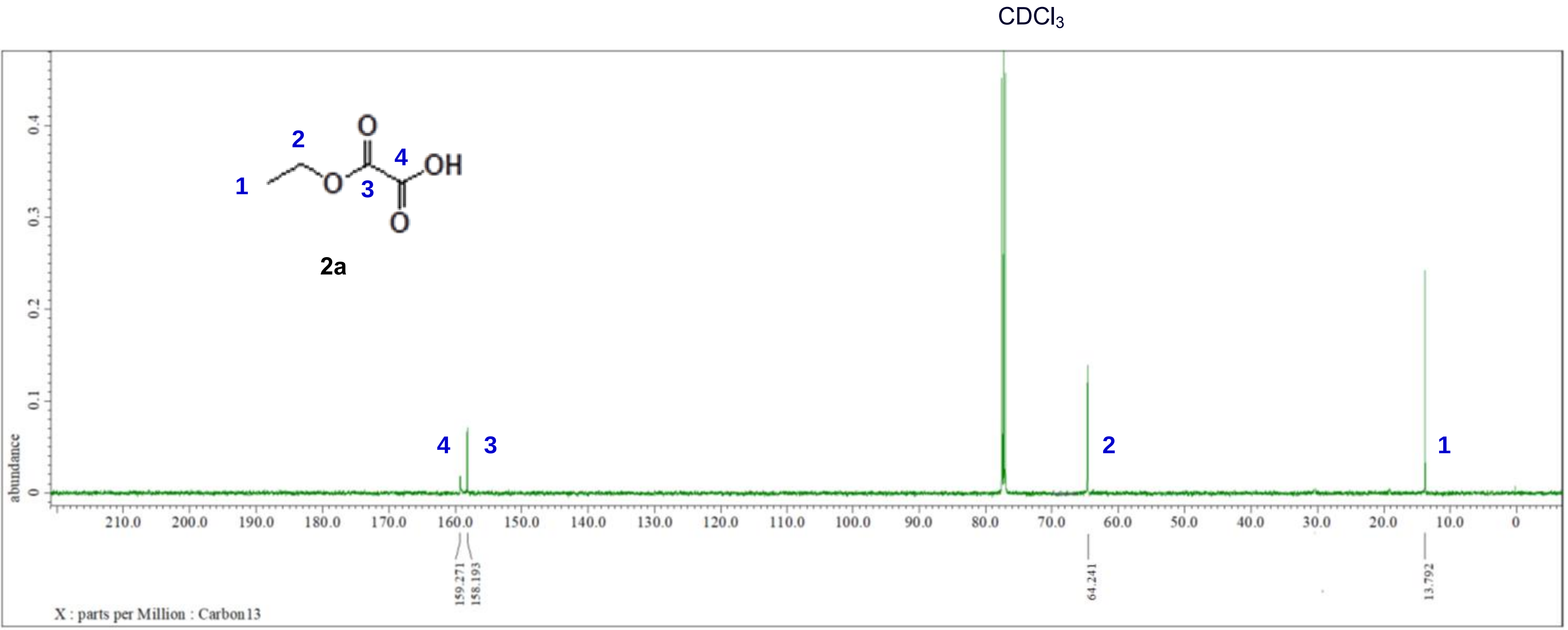
CDCl3
2a
1
2
3
4
OH
O
abundance
X : parts per Million : Carbon13
159.271
158.193
64.241
13.792
210.0
200.0
190.0
180.0
170.0
160.0
150.0
140.0
130.0
120.0
110.0
100.0
90.0
80.0
70.0
60.0
50.0
40.0
30.0
20.0
10.0
0
0.4
0.3
0.2
0.1

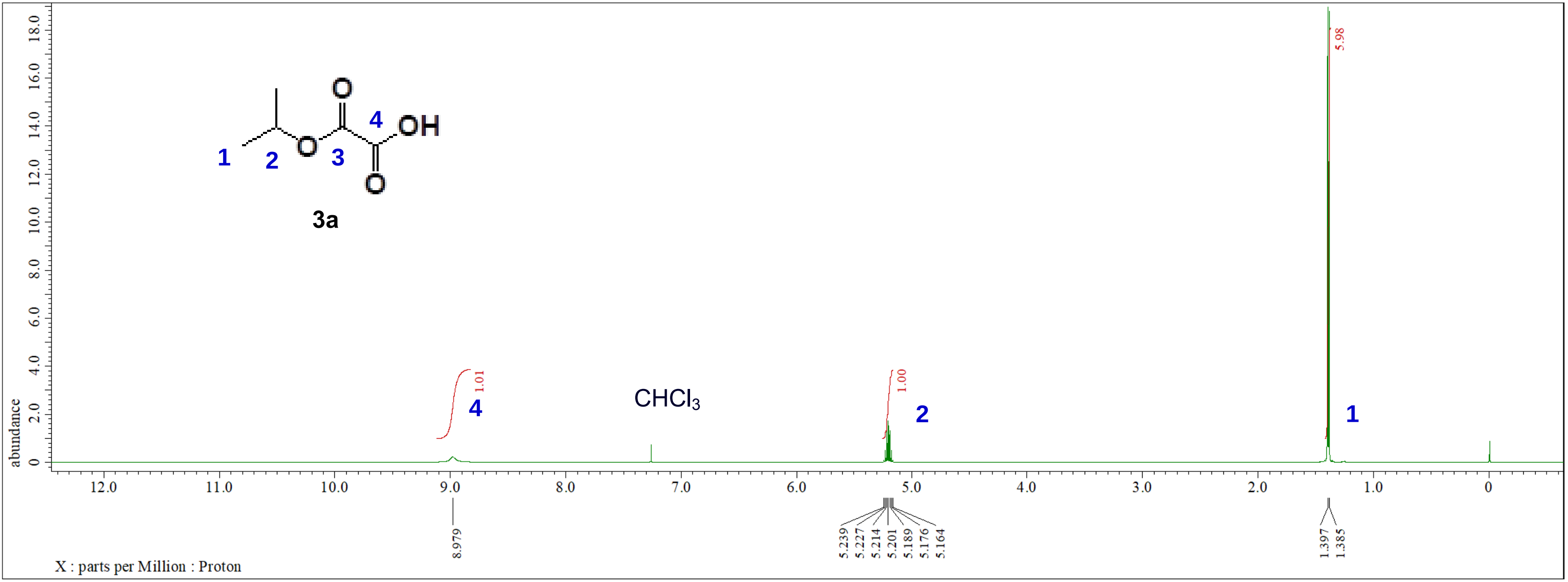
1
2
3
4
OH
3a
CHCl3
1.01
1.00
5.98
8.979
5.239
5.227
5.214
5.201
5.189
5.176
5.164
1.397
1.385
abundance
X : parts per Million : Proton

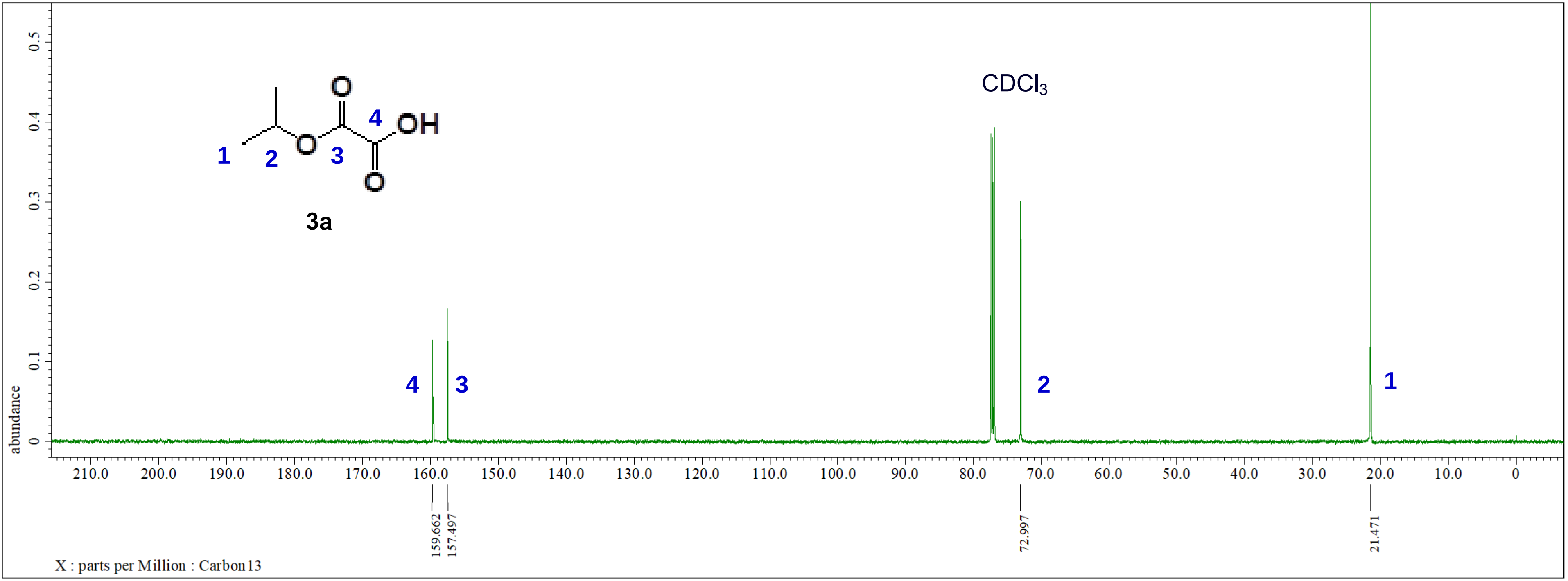
1
2
3
4
OH
O
O
O
3a
CDCl3
4
3
2
1
abundance
0.5
0.4
0.3
0.2
0.1
0
210.0
200.0
190.0
180.0
170.0
160.0
150.0
140.0
130.0
120.0
110.0
100.0
90.0
80.0
70.0
60.0
50.0
40.0
30.0
20.0
10.0
0
159.662
157.497
72.997
21.471
X : parts per Million : Carbon13

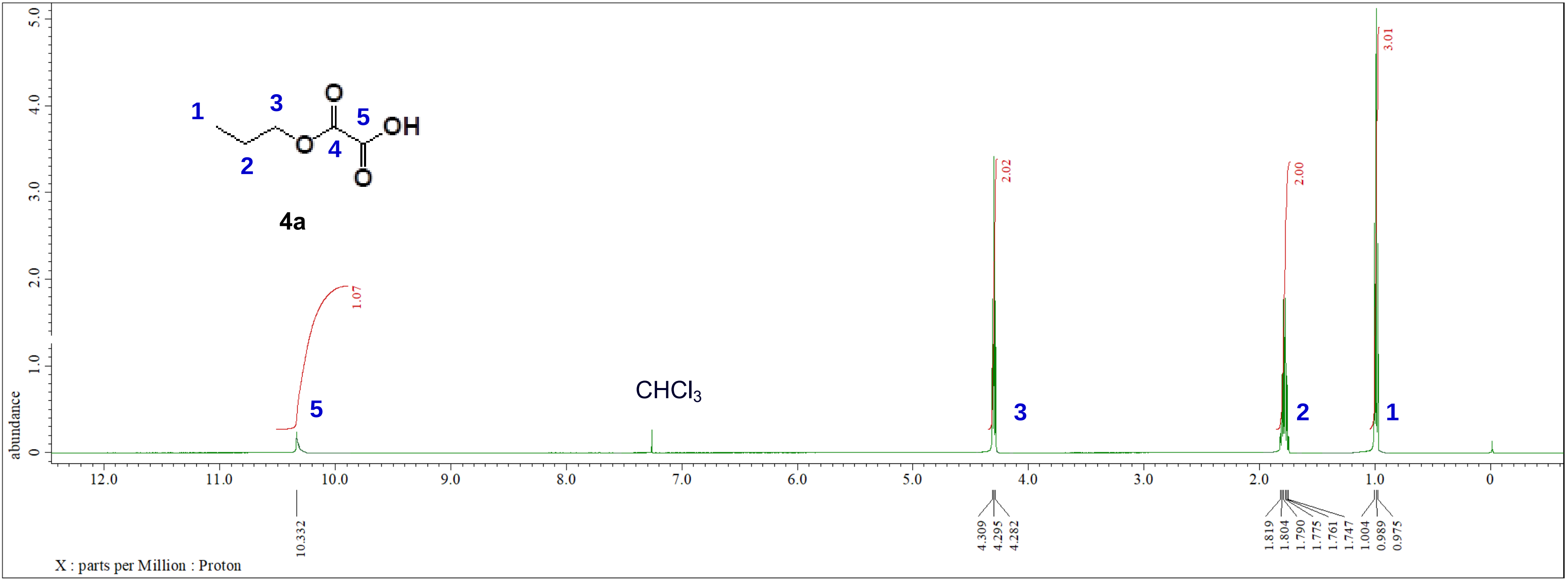

1
3
5
2
4
OH
O
O
4a
5.0
4.0
3.0
2.0
1.0
0
abundance
1.07
3.01
2.02
2.00
CHCl3
5
3
2
1
12.0
11.0
10.0
9.0
8.0
7.0
6.0
5.0
4.0
3.0
2.0
1.0
0
10.332
4.309
4.295
4.282
1.819
1.804
1.790
1.775
1.761
1.747
1.004
0.989
0.975
X : parts per Million : Proton

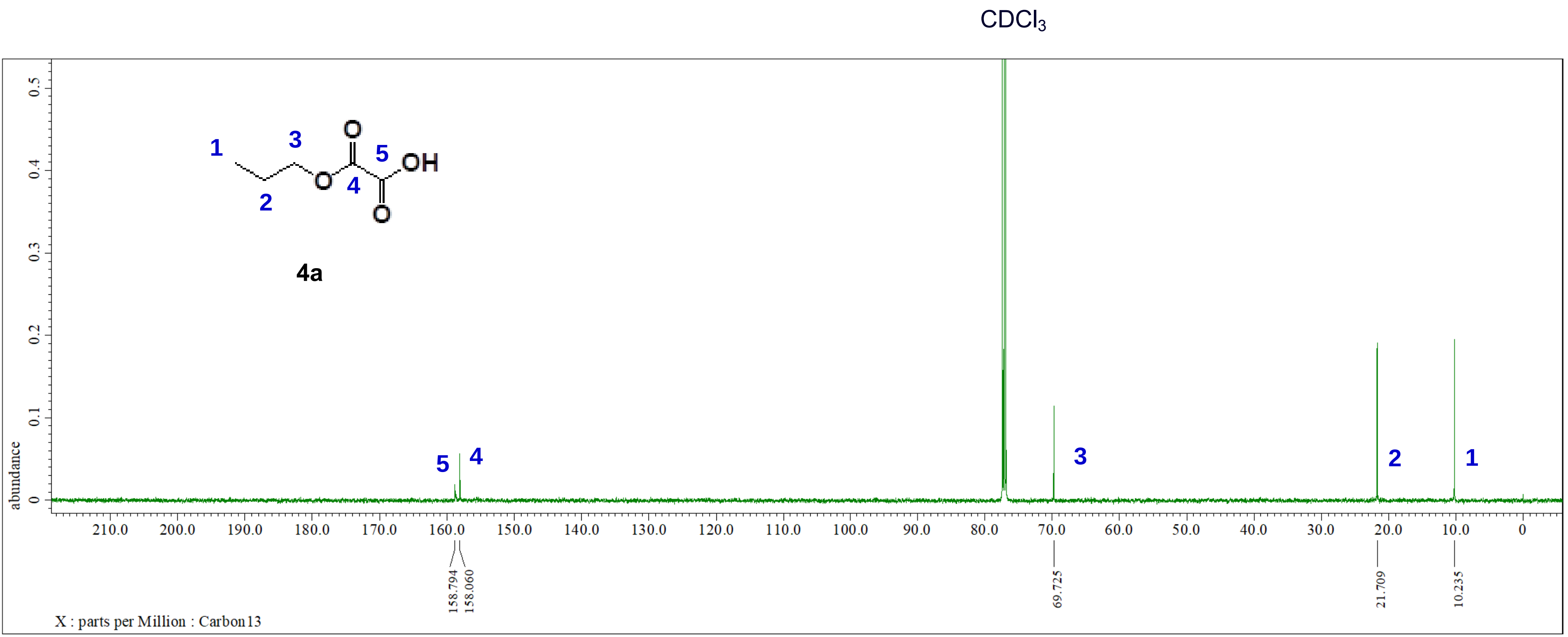
CDCl3
4a
OH
O
1
2
3
4
5
abundance
0
0.1
0.2
0.3
0.4
0.5
210.0
200.0
190.0
180.0
170.0
160.0
150.0
140.0
130.0
120.0
110.0
100.0
90.0
80.0
70.0
60.0
50.0
40.0
30.0
20.0
10.0
0
158.794
158.060
69.725
21.709
10.235
X : parts per Million : Carbon13

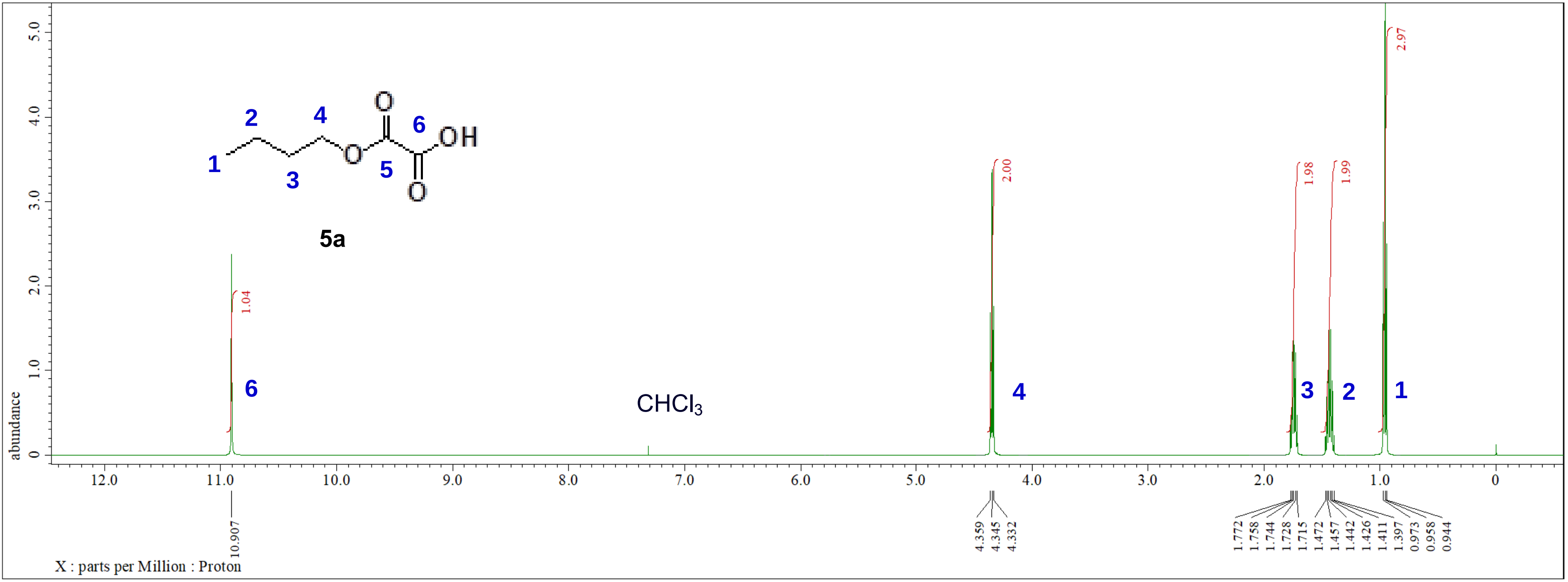

5a
6
CHCl$_3$
4
3
2
1
1.04
2.00
1.98
1.99
2.97
10.907
4.359
4.345
4.332
1.772
1.758
1.744
1.728
1.715
1.472
1.457
1.442
1.426
1.411
1.397
0.973
0.958
0.944
abundance
X : parts per Million : Proton

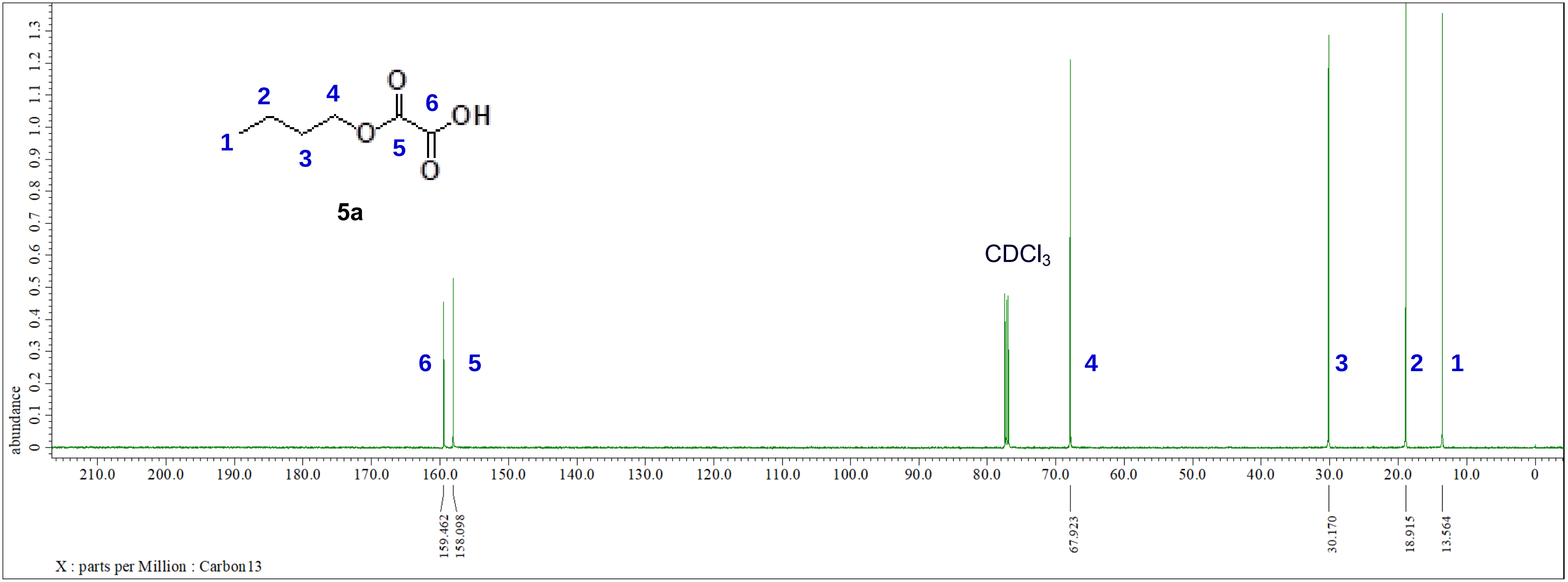

5a
CDCl$_3$
6
5
4
3
2
1
159.462
158.098
67.923
30.170
18.915
13.564
abundance
X : parts per Million : Carbon13
